# Scientists’ warning – The outstanding biodiversity of islands is in peril

**DOI:** 10.1016/j.gecco.2021.e01847

**Published:** 2021-11

**Authors:** José María Fernández-Palacios, Holger Kreft, Severin D.H. Irl, Sietze Norder, Claudine Ah-Peng, Paulo A.V. Borges, Kevin C. Burns, Lea de Nascimento, Jean-Yves Meyer, Elba Montes, Donald R. Drake

**Affiliations:** a Island Ecology and Biogeography Group, Instituto Universitario de Enfermedades Tropicales y Salud Pública de Canarias (IUETSPC), Universidad de La Laguna (ULL), 38200 La Laguna, Canary Islands, Spain; b Biodiversity, Macroecology & Biogeography, University of Göttingen, 37077 Göttingen, Germany; c Biogeography and Biodiversity Lab, Institute of Physical Geography, Goethe-University, 60438 Frankfurt, Frankfurt am Main, Germany; d Leiden University Centre for Linguistics, 2300 RA Leiden, Netherlands; e UMR PVBMT, Université de La Réunion, 97410 Saint-Pierre, La Réunion, France; f Centre for Ecology, Evolution and Environmental Changes (cE3c)/Azorean Biodiversity Group and Universidade dos Açores, Faculty of Agriculture and Environment, 9700-042 Angra do Heroísmo, Açores, Portugal; g School of Biological Sciences, Victoria University of Wellington, 6140 Wellington, New Zealand; h Délégation à la Recherche, Government of French Polynesia, 98713 Papeete, French Polynesia; i Department of Zoology, Faculty of Biological Sciences, University of Valencia, 46100 Burjassot, Valencia, Spain; j School of Life Sciences, University of Hawai]i, 96822 Honolulu, Hawai]i, USA

**Keywords:** Extinction, Insularity, Biodiversity, Human impact, Threatened species, Urgent actions

## Abstract

Despite islands contributing only 6.7% of land surface area, they harbor ~20% of the Earth’s biodiversity, but unfortunately also ~50% of the threatened species and 75% of the known extinctions since the European expansion around the globe. Due to their geological and geographic history and characteristics, islands act simultaneously as cradles of evolutionary diversity and museums of formerly widespread lineages—elements that permit islands to achieve an outstanding endemicity. Nevertheless, the majority of these endemic species are inherently vulnerable due to genetic and demographic factors linked with the way islands are colonized. Here, we stress the great variation of islands in their physical geography (area, isolation, altitude, latitude) and history (age, human colonization, human density). We provide examples of some of the most species rich and iconic insular radiations. Next, we analyze the natural vulnerability of the insular biota, linked to genetic and demographic factors as a result of founder events as well as the typically small population sizes of many island species. We note that, whereas evolution toward island syndromes (including size shifts, derived insular woodiness, altered dispersal ability, loss of defense traits, reduction in clutch size) might have improved the ability of species to thrive under natural conditions on islands, it has simultaneously made island biota disproportionately vulnerable to anthropogenic pressures such as habitat loss, overexploitation, invasive species, and climate change. This has led to the documented extinction of at least 800 insular species in the past 500 years, in addition to the many that had already gone extinct following the arrival of first human colonists on islands in prehistoric times. Finally, we summarize current scientific knowledge on the ongoing biodiversity loss on islands worldwide and express our serious concern that the current trajectory will continue to decimate the unique and irreplaceable natural heritage of the world’s islands. We conclude that drastic actions are urgently needed to bend the curve of the alarming rates of island biodiversity loss.

"*In many islands the native productions are nearly equalled or even outnumbered by the naturalised: and if the natives have not been actually exterminated, their numbers have been greatly reduced, and this is the first stage towards extinction."* (Darwin, 1859, p.380).

## 1 Scientists’ Warning initiative for island biodiversity

Scientists worldwide are becoming increasingly alarmed at the scale and severity of human impacts on our planet (Vitousek et al., 1997, Díaz et al., 2019). As a result, many argue for immediate action to reduce or reverse anthropogenic impacts on the world around us (Díaz et al., 2019; Secretariat of the Convention on Biological Diversity, 2020; WWF, 2020). Since the publication of the first Scientists’ Warning to Humanity (Union of concerned scientists, 1992) and its second notice (Ripple et al., 2017), 25 years later, the significance of biodiversity loss and its consequences for our own survival has been a key message. Both calls warned of the main threats to the environment globally (ozone layer depletion, unsustainable use of resources, habitat and biodiversity loss, climate change, and human population growth) and advised urgent actions (protecting and restoring natural habitats and ecological functions, regulating exploitation of threatened species, promoting sustainable use of resources, supporting environmental education, ensuring investment in nature protection, and providing equal access to education and wealth) to avoid the consequences of the last decades' trajectory and encourage a shift to a more sustainable society. Lately, this initiative has resulted in a series of warnings from different scientific communities on specific topics: invasive alien species (Pyšek et al., 2020), lake degradation (Jenny et al., 2020), insect extinction (Cardoso et al., 2020), endangered food webs (Heleno et al., 2020), freshwater biodiversity (Albert et al., 2020a), human population growth (Lidicker, 2020), and climate change (Ripple et al., 2021), among others. Islands are no exceptions to these global threats. It is widely acknowledged by scientists and international organizations that islands are outstanding by hosting a significant proportion of global biodiversity which is endangered (Bellard et al., 2014a, Bellard et al., 2014b; IPBES, 2019); yet despite the growing knowledge (Whittaker and Fernández-Palacios, 2007; Patiño et al., 2017, Russell and Kueffer, 2019, Whittaker et al., 2017) and efforts of institutions and governments worldwide (Local2030 Islands Network, 2019, Smart Islands Initiative, 2016) to protect their natural values, islands maintain their status as highly vulnerable regions that require special attention as reserves of biodiversity (Myers et al., 2000; Network of Islands and Coastal Biosphere Reserves, 2009). In this contribution, we highlight great threats to the Earth’s islands and their biodiversity: these fragile ecosystems lie at the intersection of many global threats, making the need for action especially urgent (Gillespie and Clague, 2009, Whittaker et al., 2017, Borges et al., 2019).

## 2 Islands: why are they so important?

**Fig. 1:**
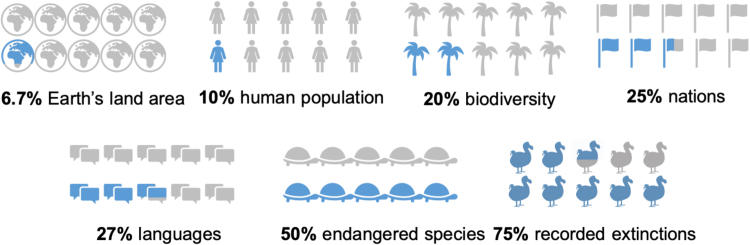
Islands’ contribution to some global statistics. For references see the text.

Islands comprise roughly 10 M km^2^ of the approximately 150 M km^2^ of emerged land in the world, thus contributing 6.67% to the world's emerging land (Sayre et al., 2018). Nevertheless, they are estimated to harbor a disproportionate 20% of the world’s biota, in addition to an extraordinary cultural and linguistic human diversity (Kier et al., 2009, Gavin and Sibanda, 2012, Tershy et al., 2015). Islands are furthermore home to about 10% of the world’s human population (Global Island Database, 2020), and one in four of the world’s 195 nations are islands or archipelagos (Fig. 1); these island nations have large exclusive economic zones with huge territorial claims to surrounding oceans. Thus, island-dwelling people are stewards of one sixth of the Earth’s surface, including many of its most endangered species and vulnerable ecosystems (Whittaker et al., 2017).

Relative to continents, islands house a disproportionate amount of the Earth’s biodiversity, yet they are especially vulnerable to anthropogenic disturbances. Oceanic islands were among the last places to be discovered and colonized by humans, yet they are some of the most severely impacted places on the planet (Whittaker et al., 2017, Russell and Kueffer, 2019, Nogué et al., 2021). We demonstrate here that extinction rates are disproportionately high on islands and that islands collectively serve as a warning for the uncertain future for the world’s biodiversity in the Anthropocene (Ricketts et al., 2005, Cardillo et al., 2006, Johnson et al., 2017, Díaz et al., 2020). Here, we aim at synthesizing existing scientific knowledge of the contribution of islands to global biodiversity and of the importance of conserving that biodiversity.

### 2.1 Island types and their physical environments

We refer to marine islands, defined as landmasses smaller than Australia surrounded by ocean. We acknowledge that the geological origin of marine islands and the classification of physical island types is complex (Ali, 2017, Ali, 2018), but oceanic islands in the strictest sense are either volcanic islands, i.e., islands that emerged as volcanoes from the seabed and have never been connected to a continent, or raised limestone islands (e.g., Makatea, ʻEua, or Niue). Iconic examples of volcanic islands include Hawaiʻi or the Galápagos, and atolls, which represent the last stage in the life of a tropical volcanic island now represented by only a coral reef (e.g., Tuamotu or Maldives). Other marine islands include land-bridge islands, which represent continental peninsulas that, owing to interglacial sea-level rise, lost their connection to continents (e.g., Great Britain, Tasmania or Newfoundland). A third island type includes continental fragments, or micro-continents, which originated as continental land surfaces, but are now isolated in the ocean through continental drift (e.g., Madagascar or New Zealand).

Although the exact number of islands is unknown, according to the most recent geographical mapping approaches there may be more than 340,000 marine islands (Sayre et al., 2018), including 17 > 0.1 M km^2^ and 20 000 > 1 km^2^ (Weigelt et al., 2013). In addition to marine islands, there are hundreds of thousands of lacustrine (such as Manitoulin in Lake Huron) or riverine (such as Marajó in the Amazonas) islands. Finally, there may be as many as one million seamounts (Staudigel and Koppers, 2015), which rise from the seabed without reaching the ocean’s surface, but nevertheless create insular conditions in the marine realm that are different from the surrounding abyssal plains. Seamounts are important habitats for sea-life: some reach the photic zone, permitting the existence of benthic photosynthetic communities, and some even emerge above the ocean’s surface in times of lower sea levels (e.g., during glaciation periods), thereby acting as important stepping stones, facilitating dispersal of species to isolated islands. In this essay, we focus on the terrestrial biota of marine islands, leaving aside other island types.

Islands can be found in all ocean basins, at all latitudes, and consequently in all climate zones. Nevertheless, two thirds of them are located in tropical latitudes (Weigelt et al., 2013). Leaving aside Greenland (actually several islands united by an ice cap), islands vary in area by at least ten orders of magnitude, from New Guinea (0.78 M km^2^) to rocks less than 10 m^2^; and they vary in age from ca. 150 My (Madagascar) to just a few decades (e.g., Surtsey, born in 1963); in elevation, from islands with mountains exceeding 4000 m (e.g., New Guinea, Borneo, Taiwan, and Hawaiʻi) to flat atolls just centimeters above the sea level; in isolation, from more than 6000 km (Society Islands) to hundreds of meters (e.g. Anglesey, Sicily, and Sakhalin); and finally in latitude from 84° N (Oodak, Greenland, the world’s northernmost emerged land) to 81° S (Berkner Island, Antarctica) (Fernández-Palacios, 2010). It is noteworthy that most marine islands are geographically not very isolated, and an estimated 75% have been connected to the neighboring mainland during Pleistocene sea-level low-stands (Weigelt et al., 2013).

Islands are renowned for the many and diverse scientific breakthroughs that their fascinating biotas have sparked during the past 250 years, and they continue to serve as model systems for research in biogeography, ecology, evolution, and conservation (Warren et al., 2015, Patiño et al., 2017). Charles Darwin and Alfred Russel Wallace independently discovered the principles of evolution after extended travels through the island archipelagos of the world. MacArthur and Wilson’s equilibrium theory of island biogeography (MacArthur and Wilson, 1963; 1967) has become the most influential theory in biogeography, and has major relevance to other biological fields, including conservation biology (Warren et al., 2015, Whittaker et al., 2017). Peter and Rosemary Grant’s (Grant and Grant, 2008) work on the dynamic adaptation of beak size and form in Galápagos finches to variation in food sources has become a textbook example of how rapidly evolution can occur in nature. Historically, research on islands has been invaluable for the development and enrichment of many scientific disciplines.

## 3 Islands as outstanding centers of biodiversity

**Fig. 2:**
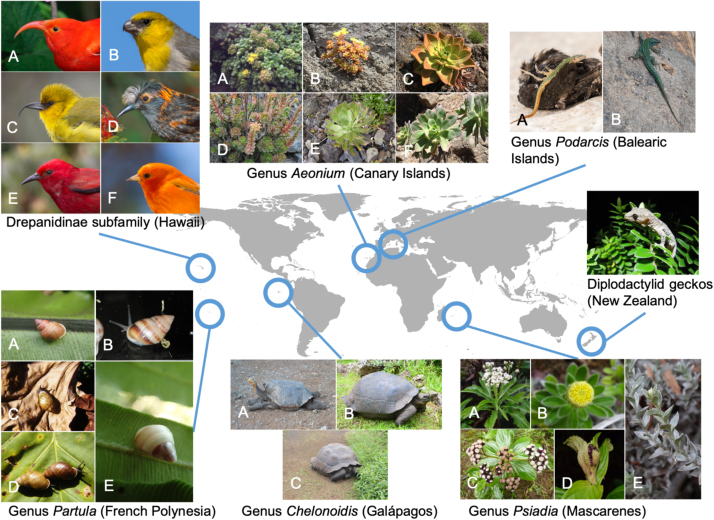
Iconic and less known examples of adaptive radiation from various islands worldwide, such as the endemic monophyletic subfamily of honeycreepers Drepanidinae from Hawaiʻi (A: *Drepanis coccinea*, B: *Loxioides bailleui*, C: *Hemignathus wilsoni*, D: *Palmeria dolei*, E: *Himatione sanguinea*, F: *Loxops coccineus*, photos by Jack Jeffrey Photography), the genus *Aeonium* from Macaronesia (A: *Aeonium lindleyi*, B: *A. sedifolium*, C: *A. nobile*, D: *A. spathulatum*, E: *A. urbicum*, F: *A. percarneum*, photos by Severin Irl), the genus *Podarcis* of wall lizards from the Balearic Islands (A: *Podarcis pityusensis schreitmulleri*, B: *P. pityusensis formenterae*, photos by Baravi Thaman and Jordi Serapio), the Diplodactylid geckos from New Zealand (*Woodwarthia maculate*, photo by Susan Keall), the genus *Psiadia* from the Mascarenes (A: *Psiadia laurifolia*, B: *P. callocephala*, C: *P. boivinii*, D: *P. insignis*, E: *P. argentea*, photos by Arnaud Rhumeur and Alexis Gorissen), the genus *Chelonoidis* of giant tortoises from Galápagos (A: *Chelonoidis hoodensis*, B: *C. porteri*, C: *C. guentheri*; photos by Anna Walentowitz) and the genus *Partula* of tree snails from French Polynesia (A: *Partula otaheitana*, B: *P. labrusca*, C: *P. navigatoria*, D: *P. suturalis vexillum*, *P. tohiveana*, photos by Justin Gerlach). For details see Table 1.

Among the islands of the world, volcanic islands, micro-continents, and large tropical land-bridge islands (such as New Guinea, Sumatra, Borneo, and Java) make disproportionately large contributions to global biodiversity. Much of this biodiversity results from cases of so-called ‘explosive radiation events’, which typically result from lineages colonizing an isolated island, then responding to dynamic, heterogeneous environments, which are thought to provide ample opportunities for adaptive (Losos and Ricklefs, 2009) or non-adaptive (Czekanski-Moir and Rundell, 2019) diversification (Table 1; Fig. 2).

**Table 1:** Examples of radiation events of selected island taxa, contributing significantly to the world’s biodiversity.

Island/archipelago	Taxa	Species number	References
Caribbean islands	*Anolis* lizards	≃ 155	Losos (2009)
	*Coccothrinax* palms	50	Dransfield et al. (2008)
Galápagos	*Bulimulus* land snails	70	Chambers (1991)
	Darwin’s finches (Geospizinae)	15	Grant & Grant (2008)
	*Scalesia* (Asteraceae)	15	Fernández-Mazuecos et al. (2020)
Hawaiʻi	Drosophilid flies	≃ 1000	O’Grady & Desalle (2018)
	Trigonidiinae crickets	173	Shaw (1995)
	Lobeliads (Campanulaceae)	≃ 126	Givnish et al. (2009)
	*Cyrtandra* (Gesneriaceae)	60	Kleinkopf et al. (2019)
	*Hylaeus* bees	60	Magnacca (2007)
	Honeycreepers (Drepanidinae)	≃ 60	Pratt (2005)
	*Tetragnatha* spiders	56	Cotoras et al. (2018)
	Silverswords (Asteraceae)	28	Baldwin & Sanderson (1998)
Macaronesia	*Laparocerus* beetles	236 (sp. & ssp.)	Machado et al. (2017)
	*Napaeus* snails	77	Alonso & Ibañez (2015)
	*Dysdera* spiders	64	Arnedo et al. (2000); Macías-Hernández et al. (2016); Crespo et al. (2020)
	*Tarphius* beetles	64	Emerson & Oromí (2005); Borges et al. (2008); Borges et al. (2017)
	*Aeonium* clade (Crassulaceae)	60	Arechavaleta et al. (2005); Borges et al. (2008); Arechavaleta et al. (2010); Borges et al. (2010)
	*Sonchus* clade (Asteraceae)	35	Cho et al. (2019)
	*Echium* (Boraginaceae)	29	García-Maroto et al. (2009); Carvalho et al. (2010)
Madagascar	*Dombeya* (Malvaceae)	≃ 175	Skema (2012)
	*Dypsis* palms	170	Dransfield & Beentje (1995)
	*Psychotria* (Rubiaceae)	143	Taylor (2020)
	Lemurs (Lemuroidea)	117	Herrera (2017)
	*Pandanus* (Pandanaceae)	≃ 100	Callmander & Laivao (2003)
	Vangas birds (Vangidae)	22	Jønsson et al. (2012)
Mascarenes	*Cratopus* (Curculionidae)	80	Kitson et al. (2013)
	*Psiadia* (Asteraceae)	27	Strijk et al. (2012)
	Dombeyoideae (Malvaceae)	23	Le Péchon et al. (2013)
New Caledonia	*Phyllanthus* (Phyllanthaceae)	116	Munzinger et al. (2016)
	*Psychotria* (Rubiaceae)	85	Barrabé et al. (2014)
	Lygosominae skinks	≃ 50	Smith et al. (2007)
	Diplodactylid geckos	36	Skipwith et al. (2019)
New Guinea	Orchidaceae	≃ 2800	Vollering et al. (2016)
	Microhylid frogs (Asterophryinae)	> 200	Rivera et al. (2017)
	Birds of paradise (Paradisaeidae)	≃ 40	Heads (2001)
New Zealand	*Hebe* (Plantaginaceae)	120	Bayly & Kellow (2006)
	Cicadas (Cicadidae)	55	Arensburger et al. (2004)
	*Olearia* (Asteraceae)	44	https://www.nzpcn.org.nz/flora/species
	Diplodactylid geckos	36	Nielsen et al. (2011)
French Polynesia	*Mecyclothorax* ground beetle	101	Liebherr (2013)
	*Miocalles* flightless weevils	67	Paulay (1985)
	*Partula* tree snails	60	Lee et al. (2014)
	*Bidens* (Asteraceae)	24	Knope et al. (2020)
Sulawesi	*Tylomelania* freshwater gastropods	35	Von Rintelen & Glaubrecht (2005)
Ogasawara	*Mandarina* snails	17	Chiba (1999)
Philippines	Orchidaceae	≃ 1200	Buenavista (2017)
	Earthworm mice (*Apomys*)	30	Heaney et al. (2016)
	Cloud rats (Murinae)	18	Heaney et al. (2016)
Fiji	*Psychotria* (Rubiaceae)	77	Heads (2006)
	*Strumigenys* Trap-jaw ants	23	Sarnat et al. (2019)
	*Homalictus* bees	22	Groom et al. (2013)
Juan Fernández	*Dendroseris* (Asteraceae)	11	Sang et al. (1994)

The approximate number of endemic species is given and includes both extant and extinct species. All references in this table can be found in Table S1 in the Online Supporting Information.

### 3.1 Oceanic islands as museums of biodiversity

Volcanic islands and, particularly, micro-continents can act as ‘museums’ of biodiversity, where lineages or entire ecosystems—long since extirpated from the continents—still form part of the extant insular biota (Cronk, 1997). The role of islands as refugia for such paleoendemic taxa results from several phenomena. First, the buffer effect exerted on islands by the surrounding oceans provides them with milder climates than those experienced on the continents at similar latitudes. This effect has been especially important during episodes of climatic change in the Pleistocene. Secondly, many oceanic islands have a steep topography and attain high elevations, allowing short-distance altitudinal migration by species to track suitable climatic conditions. Again, this feature is particularly important when climate changes. Thirdly, the reduced interspecific competition on many oceanic islands can allow the survival of forms that have since been outcompeted by evolutionary novelties on continents. Finally, small islands and islets located off the mainland or larger islands can act as refugia for species extinguished by humans on the mainland through hunting or the introduction of invasive alien species. Such examples of insular paleoendemism include laurel forest tree species (*Apollonias*, *Heberdenia, Ocotea, Persea, Pleiomeris, Persea* and *Visnea*) in Macaronesia (Fernández-Palacios et al., 2019), the Galápagos (*Chelonoidis nigra* complex) and Aldabra (*Aldabrachelys gigantea*) giant tortoises, the tuatara (*Sphenodon punctatus*) on islets off New Zealand, New Caledonia’s nearly flightless cagou (*Rhynochetos jubatus*) and the shrub *Amborella trichopoda*, sister to all other extant angiosperms and the sole survivor of a Late Jurassic lineage (*c*. 160 Mya) (Pillon and Buerki, 2017), and *Lactoris fernandeziana,* a relic flowering plant species from Juan Fernández, that constitutes the sole extant member of the monotypic order Lactoridales, a very primitive taxon, ca. 90 My old, which could have played a role in angiosperm evolution at the transition between dicots and monocots (Stuessy et al., 1998).

### 3.2 Islands as evolutionary cradles

Although most lineages on islands do not diversify, those that do commonly contribute disproportionately to diversity and endemism (Table 1, Fig. 2). For this reason, oceanic archipelagos have been labeled as evolutionary arenas (Nürk et al., 2020), in the sense that new endemic species, generated from few colonization events, continuously evolve there. Several archipelago characteristics create conditions that promote the phenomenon of intra-archipelagic speciation including: i) natural fragmentation and spatial isolation of the constituent islands; ii) long-term isolation from the mainland source pool; iii) high elevations of volcanic islands and consequent generation of high habitat heterogeneity; iv) complex intra-island landscape heterogeneity created by geodynamical processes, particularly once erosion begins to dominate; v) sequential emergence of islands and the old overall age of hotspot archipelagos; and vi) eustatic sea-level changes in the Pliocene-Pleistocene that have repeatedly reshaped islands’ and archipelagos’ geographies (Whittaker et al., 2008, Weigelt et al., 2016).

The combination of these factors facilitates numerous ecological, biogeographic, and evolutionary processes, resulting in high speciation rates in these evolutionary arenas (Baldwin and Sanderson, 1998; Losos and Ricklefs, 2009). Within a single high-elevation island, adaptive radiation may generate new species that exploit diverse habitats and resources from the coast to the summit. If the island is heavily dissected by erosion but still retains relatively high elevation, then intra-island vicariance among different ravines or slopes will contribute to intra-island diversification as well. Vicariance may also occur when island fusion and fission (for example, resulting from the sea-level changes) happens repeatedly (Weigelt et al., 2016). Moreover, topographic complexity and isolation with increasing elevation further enhance speciation rates at high elevations (Steinbauer et al., 2016), producing high concentrations of narrow-ranged endemics towards higher elevations (Irl et al., 2017).

When dispersal between islands within an archipelago is frequent, the picture complicates further. Additional processes to consider include: i) the progression rule (colonization of newer islands from older ones; Wagner and Funk, 1995); ii) double invasions (a second establishment of the same ancestor, long after the first one, which by then has originated a new species; Lack, 1947); and iii) retrocolonization (the colonization, by a new species, of the island (or mainland) from which its ancestor originated; Fernández-Palacios and Whittaker (2020)). In the latter instance, islands can act as refugia and sources for continental diversity, as has been suggested for conifers that spread from New Caledonia and New Zealand to larger land masses (Condamine et al., 2017).

## 4 The natural vulnerability of insular biota

In contrast to romanticized depictions of islands as benign, idyllic environments, the intrinsic insular geologic features make island life less than ideal. Volcanic activity may either destroy entire islands or sterilize large areas, and gravitational land-slides can displace significant portions of the island area within minutes, while the associated tsunamis can strike neighbor islands, destroying their coastal and lowland ecosystems. Magma chamber collapses, earthquakes, seaquakes, and hurricanes also take a significant toll on the insular biota. Finally, islands can vanish below sea level temporarily, due to marine transgressions, or permanently, due to subsidence (Menard, 1986).

Nevertheless, these destructive processes simultaneously create ecological opportunities for new colonizers, thus promoting colonization and, in the long run, diversification and endemicity. As these catastrophic geological events are natural, very rare, and act over very long timescales, we will not comment on them further. Instead, we will focus on some demographic, genetic, and evolutionary characteristics of island populations and species that make insular biota intrinsically vulnerable to population decline and extinction.

As a result of isolation, limited area, and natural fragmentation of islands and archipelagos, their insular biota displays several geographic, demographic, and genetic features that make them naturally vulnerable to multiple processes that erode biodiversity, even in the absence of human activities (Frankham et al., 2002). Those features are mainly related to the small and naturally fragmented distribution ranges of island populations of more widely distributed species, or of insular endemics when compared with their closest mainland relatives (James et al., 2016). Small distribution ranges imply (except for those species that exhibit density compensation) smaller population sizes and smaller effective population sizes, and higher risks of stochastic or demographic collapse or of inbreeding depression. The founding of island populations also implies lower (and unusual) genetic diversity (Frankham et al., 2002) (Table 2).

**Table 2:** Factors contributing to the natural vulnerability of insular biota (based in Frankham et al., 2002).

Insular condition	Consequence on their vulnerability
Very small distribution range (endemics are usually Single Island Endemics - SIEs)	Higher extinction risk due to stochastic events (volcanism, landslides, etc.)
	Lower population size (unless density compensation)
Naturally fragmented distribution range	High genetic differentiation at a population level, meaning that extirpations imply genetic heritage loss (populations, and not species, as operational conservation units)
Low population sizes (N)	Low effective population size (N_e_)
	Risk of stochastic demographic collapse
Few populations/ Few individuals per population	Genetic drift controls speciation, implying a non-adaptive path that creates species not fitting in their environment, promoting inbreeding depression and finally population collapse
Population origin through founder event	Population bottleneck resulting in lower and singular genetic diversity

**Fig. 3:**
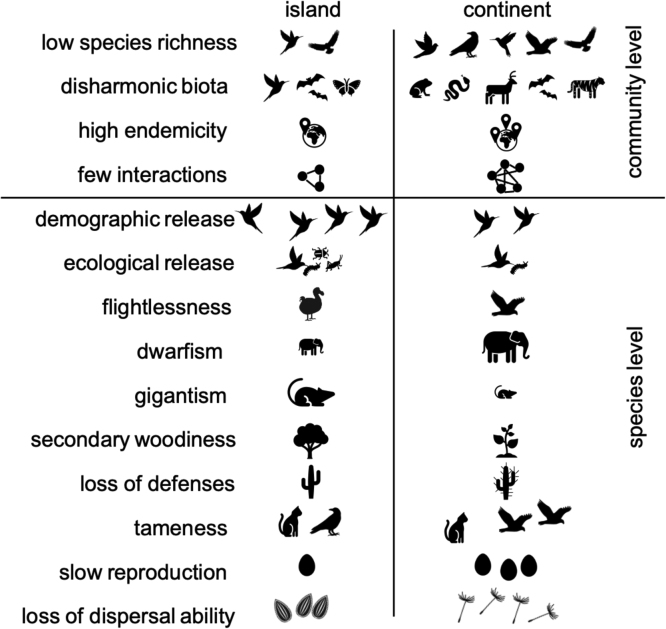
Some typical characteristics of island species and communities that make them different from continental ones.

Furthermore, the evolution of species in isolation often leads to the emergence of a series of very peculiar characteristics, referred to as insular syndrome(s) (Adler and Levins, 1994). Island biota often possess characteristics that increase their fitness in pristine island environments, including size shift (gigantism or dwarfism) (Foster, 1964, Lomolino et al., 2012, Lomolino et al., 2013), flightlessness (Carlquist, 1965, Carlquist, 1974, Roff, 1991), altered dispersal ability in plants (Carlquist, 1974, Cody and Overton, 1996, Burns, 2019), derived insular woodiness (Darwin, 1859, Wallace, 1878 Carlquist, 1965, Carlquist, 1974, Lens et al., 2013; Burns, 2019), loss of defense traits against herbivores (Carlquist, 1974, Bowen and Van Vuren, 1997, Burns, 2019), reduced defensive behaviour (Stamps and Buechner, 1985); tameness (Cooper et al., 2014; Brock et al., 2015); reduced clutch size (Covas, 2012), or trend towards dioecy (Sakai et al., 1995, Schlessmann et al., 2014), among others. Importantly, these same characteristics exacerbate their vulnerability to humans and their introduced biota in disturbed island environments (Table 3, Fig. 3). Another issue, although not an evolutionary innovation, is the fact that the persistent isolation of island species results in a lack of contact with diseases, or disease vectors, that are frequent on continents, leading to island biota being highly vulnerable to the arrival of novel diseases and disease vectors associated with species introduced by humans (Ricklefs and Bermingham, 2002).

**Table 3:** Reasons of vulnerability after human colonization of the evolutionary innovations related to island syndromes (adapted from Fernández-Palacios, 2019).

Evolutionary innovation emerging in insular contexts (island syndromes)	Reasons of vulnerability after human colonization
Gigantism	Bigger reward for hunting, source of meat
Dwarfism	Decrease of deterrence power/fierceness, facilitating hunting or predation by introduced predators
Insect and avian flightlessness	Incapacity of escaping introduced predators
Loss of dispersibility in plants	Incapacity of escaping habitat destruction
Diminution of clutch size	Less recovery potential during disturbance
Diminution of defensive behaviour / Tameness	Naive behaviour against hunting/predation
Insular secondary woodiness	Slower regeneration rate making woody species more susceptible to human-associated disturbances. Logging target for firewood, weapons, tools
Trend towards secondary (functional) dioecy	Difficulty of mating in precarious demographic conditions
Loss of plant defences against mammalian herbivory	Increased palatability of island plant species to introduced mammalian herbivores
Other isolation related issues	
Lack of contact with diseases/pathogens	Vulnerability against imported diseases

## 5 Human impacts on islands

Hominins have been roaming on islands since at least the early Pleistocene, and *Homo sapiens* for at least 50 Ka. However, the impact of these early dwellers on the biota of the islands they inhabit, was moderate and, in any case, difficult to disentangle from environmental changes (Louys et al., 2021). It was not until the beginning of the current interglacial that changes in subsistence strategies (hunter-gatherers to herders and farmers), political organization, technology, dispersal (i.e. invention of navigation), demography, and behavior visibly affected island ecosystems, especially those of oceanic islands, which, owing to their isolation were the very last frontiers of human expansion throughout the world. Many of the oceanic archipelagos were colonized only during the last few millennia (Caribbean, Madagascar, Balearics, Canaries, remote Melanesia, and Western Polynesia), the last millennium (New Zealand, Hawaiʻi, Eastern Polynesia, and Iceland), or even just the last few centuries (Madeira, Azores, Cabo Verde, Mascarenes, Galápagos, and Tristan da Cunha). Thus, the Late Pleistocene megafaunal extinction on the continents that began with the arrival of humans in Australia and the Americas (Stuart, 2015) marks a final, still ongoing Holocene episode on the oceanic islands of the world, where insular flora and fauna (such as Caribbean sloths, New Zealand moa, Malagasy elephant bird and giant lemurs, Mascarene dodo, Hawaiian moa nalo and nēnē nui (Anseriformes), Caribbean monk seal, or Steller’s sea cow, among many others) have been driven to extinction after human colonization (Hume, 2017, Wood et al., 2017, Nogué et al., 2021).

As shown above, extinction risks from natural causes for island biota are much higher than for continental species, even in the absence of human activities. Alarmingly, the current presence of humans on islands exacerbates the threat to island genetic heritage, biota and ecosystems, making islands hotspots of the current global biodiversity crisis (Whittaker et al., 2017, Leigh et al., 2019, Russell and Kueffer, 2019, Nogué et al., 2021).

There are at least four main causes, directly or indirectly related to human activities, that induce biodiversity erosion and species loss on islands: i) habitat loss, ii) resource overexploitation, iii) introduction of alien species, and iv) climate change. Each of these direct drivers puts tremendous pressure on biodiversity; effects are, however, aggravated when drivers act synergistically.

### 5.1 Habitat loss

Habitat loss—the destruction, degradation, modification, or fragmentation of habitats—occurs when natural environments are transformed or modified to serve human needs. It is the most significant cause of biodiversity loss globally, and on islands (Borges et al., 2019). Common types of habitat loss on islands include logging for timber, deforestation to open up land for livestock or agriculture, draining wetlands, and urban expansion.

Habitat loss on islands began with the arrival of the first island dwellers and with the species introduced by them, either intentionally (ovicaprids, pigs, dogs, chicken, etc.) or accidentally (rats, mice, invertebrates). Fire was used from the very beginning to transform ecosystems and create agricultural areas and rangeland (Burney, 1997; McWethy, 2009; Rick et al., 2013; de Nascimento et al., 2020). Very soon after subsequent European colonization, the lowlands and mid-elevation ecosystems of many islands worldwide had been almost completely transformed in order to sustain an export-oriented intensive agriculture, such as sugar cane (including vast amounts of wood needed by sugar mills; Hawaiian Islands, La Réunion, and Santo Antão), bananas (La Palma), pineapples (Philippines), olive tree (Crete, Sardinia, Sicily, and Cyprus), oil palm (Borneo and Sumatra), tea (Sri Lanka), and vanilla (Madagascar). On other islands, large swaths of native vegetation were transformed for rangeland (Azores, Hawaiʻi and New Zealand), forestry plantations (Azores and New Zealand), or the huge infrastructure (airports, harbors, resorts, golf courses, roads, etc.) needed for maintaining mass tourism (Mallorca, Gran Canaria, Tenerife, Oʻahu, Mauritius, Madeira, and Sal). Furthermore, islands have been particularly affected by economic crises following the booms and busts of different monocultures, which have been sequentially replacing each other over time (e.g., on the Canaries, from the Castilian conquest on: sugar cane, wine, natural colorants from insects growing on *Opuntia*, bananas, tomatoes, and finally, mass tourism) (Norder et al., 2020).

The transformation of natural ecosystems implies the disappearance of the original ecosystems and their associated species. On the Canaries, the laurel forest of Gran Canaria was depleted to the current 1% of its original distribution, whereas Tenerife’s thermophilous woodlands were even more severely reduced (del Arco et al., 2010). In the Azores, the original forests were substituted by rangeland and by the plantation of exotic *Cryptomeria* (Fernández-Palacios, et al., 2019b); Porto Santo, in the Madeira Archipelago, was totally deforested (Rocha et al., 2017), and Nauru, in the Central Pacific, was irreversibly degraded through the mining of its rich phosphate deposits (Gale, 2019).

Habitat loss can also cause fragmentation, which makes it difficult for species to move between habitat patches and causes additional detrimental effects such as edge effects (Laurance and Yensen, 1991). Though some habitat loss is necessary to meet human needs, when natural habitats are changed or modified the results can be very negative. Examples include the massive replacement of forest habitats by open grassland that took place in the Hawaiian Islands, which also changed landscape hydrology and fire regimes and promoted the massive extinction of forest dependent species, and the large-scale alterations of coastal habitats (including mangroves) for real estate and tourism purposes (Barton et al., 2021). While some of the negative effects of habitat loss and modification have already been manifested in biodiversity losses, considerable extinction debts (Triantis et al., 2010) and interactions with other drivers make it difficult to assess their full long-term impact. On many islands, immediate action is needed to protect the last patches of remaining native vegetation and implement effective restoration strategies.

### 5.2 Resource overexploitation

Overexploitation and the unsustainable use of living resources, including logging, hunting, fishing, and collecting, happens when biodiversity is removed faster than it can be replenished: this, over the long term, results in the extinction of species. Nevertheless, overexploitation of abiotic resources, such as the water table, can also cause extinctions, as happens for instance with the loss of springs and the consequent desiccation of the associated freshwater ecosystems, which on islands contain mainly endemic species.

Although evidence of a human role in megafauna extinction in the Mediterranean and Caribbean islands is still controversial, there is overwhelming evidence of overexploitation by humans being the leading cause of the megafaunal extinctions in Madagascar and the Pacific islands (Turvey, 2009). Moa (Dinornithidae) and Elephant-birds (*Aepyornis*), the largest birds ever existing, along with many other large-bodied, charismatic fauna, were hunted to extinction by Maori and Malagasy within a relatively short time after colonization, much as later Portuguese and Dutch sailors did with the Mauritian Dodo (Cheke and Hume, 2008) or Russian fur-hunters with Steller’s sea cow on Bering island (Anderson, 1995).

Hunting for scientific collections has also driven some species to extinction, such as the spectacular New Zealand huia (*Heteralocha acutirostris*) (Johnson and Stattersfield, 1990). Another sad example is the Gran Canaria endemic blue chaffinch (*Fringilla polatzeki*), which was first discovered to science only in 1905. Immediately after its discovery, natural history museums from all over Europe sent naturalists to gather specimens of the newly discovered species; within a few years, more than 100 individuals were collected, with one person responsible for collecting some 76 birds between January and April of 1909. This depletion had a lasting impact on the bird population, and today the species is designated as critically endangered; with an estimated population size of around 250 individuals, it is considered the most endangered bird species in the European Union (Rodríguez and Moreno, 2004).

### 5.3 Invasive alien species

*Invasive alien species* (IAS)—species that have overcome biogeographic barriers through the deliberate or accidental introduction by humans, and which have subsequently invaded native ecosystems and had negative ecological impacts—are a major cause of biodiversity loss on islands. IAS may also cause economic or environmental damage, or adversely affect human health (Sax and Gaines, 2008). The important role IAS play as drivers of biodiversity changes has been known to science since at least the time of Charles Darwin (1859) (see epigraph). IAS are harmful to native biodiversity in a number of ways; for example as predators, parasites, vectors of disease, disruptors of mutualistic relationships, or competitors for habitat and resources (Williamson, 1996). In many cases, IAS that come to an island face few or no parasites, predators, or herbivores in their new environment, so their population size is often not controlled by top-down processes (enemy release hypothesis; Keane and Crawley, 2002). Some IAS thrive in disturbed or degraded habitats and can thus work in conjunction with or augment other environmental stressors.

Some of the most detrimental IAS have been intentionally introduced, such as crops or livestock (e.g. goats, sheep, and pigs), pets (e.g. cats and birds), ornamentals (e.g. *Hedychium gardnerianum* and *Lantana camara*), biological controls (e.g. cane toads and mongooses), or game species (e.g. foxes and deer). For plants, important reservoirs of IAS are domestic and botanical gardens, where the majority of the global naturalized alien ﬂora is grown (van Kleunen et al., 2018). Introductions can also be accidental, such as when species are introduced through ballast water or in cargo containers (brown tree-snake *Boiga irregularis* introduced to Guam, coconut rhinoceros beetle *Oryctes rhinoceros*, and numerous species of ants). The main vectors for IAS are trade, transport, travel, and tourism, all of which have increased immensely in recent years (Seebens et al., 2018). Recent studies have identified islands as global hotspots of IAS, resulting in increased extinction risk for vulnerable island biota (Dawson et al., 2017). The introduction of IAS has even led to reversal of fundamental island biogeographic principles, e.g., the well-known species-isolation relationship is reversed for alien species due to increased island vulnerability as a result of lower diversity and lower biotic resistance of the recipient island biota with increasing remoteness (Moser et al., 2018).

One outstanding example of IAS impacts is the Pacific brown tree-snake in Guam, which led to the documented extinction of ten species of birds, bats and reptiles (Rodda et al., 1999). Another is the intentional introduction of the predatory rosy-wolf snail *Euglandina rosea* to control a second invasive alien snail, the giant African snail *Lissachatina fulica*, and which inadvertently caused the extinction of 28 of the 55 species of the endemic *Partula* tree snails of the Society Islands (Gerlach, 2016)*.* The *Hedychium gardnerianum* invasion in the Hawaiian and Azorean islands is completely changing the soil cover of wet forests, with huge impacts on bryophytes, ferns, and particularly invertebrates (Borges et al., 2017). The massive invasion of Tahitian rainforest and cloud forest by the small tree *Miconia calvescens* has imperiled half of the 100 endemic plants (Meyer and Florence, 1996).

Rodents (especially *Rattus* spp. and *Mus musculus*) are among the earliest and perhaps the most widely established and destructive IAS on islands, where they have great impacts as predators, granivores, and herbivores (Towns et al., 2006, Drake and Hunt, 2009, Carpenter et al., 2020). Black rats (*Rattus rattus)* have been blamed for the extinction of six endemic species of monarch flycatchers (*Pomarea* spp.) in the Marquesas and the Society Islands (Thibault et al., 2002) and pushed two other species to the brink of extinction.

Introduced herbivores have also led to massive alterations of island habitats. In particular, generalist mammalian herbivores such as feral goats (*Capra hircus*) or the European rabbit (*Oryctolagus cuniculus*) are directly threatening island floras, because endemic species are preferentially consumed due to a relative lack of chemical or physical defense traits against mammalian herbivores (e.g. Cubas et al., 2019). Consequently, on many islands, palatable plant species have been dramatically reduced or are found only in inaccessibly steep sites. Introduced herbivores also indirectly threaten many other taxonomic groups (mammals, birds, invertebrates, etc.) as a result of the alteration and destruction of natural habitat, as has been extensively shown for feral goats and other introduced herbivores on the Galápagos Islands (Coblentz, 1978; Tye, 2006). In many cases, restoration of natural habitat is possible if quick and decisive action is taken. Promising examples include feral goat eradications from at least 120 islands worldwide (Campbell and Donlan, 2005), or as a last resort, translocations of threatened species to IAS-free, fenced areas (Ewen et al., 2011).

Invasive alien species are not independent of each other in their ecological impacts. Islands frequently suffer from invasional meltdown (Simberloff and Von Holle, 1999), a process that occurs when IAS interact synergistically, facilitating each other’s spread or intensifying each other’s impacts. The Macaronesian faya tree (*Morella faya*) in Hawaiʻi is a good example. Its success is associated with its ability to fix nitrogen; this has quadrupled the availability of nitrogen in nitrogen-limited, early-successional ecosystems, altering their successional trajectories and facilitating subsequent invasions, mediated by the presence of feral pigs and several continental weeds that are better adapted to higher nitrogen conditions than are the native plants (Vitousek and Walker, 1989). Other examples include the mutualistic interaction between the yellow crazy ant *Anoplolepis gracilipes* and scale insects on Christmas Island (O’Dowd et al., 2003) and the introduced birds and invasive alien plants they disperse on Oʻahu, Hawaiʻi (Vizentin-Bugoni et al., 2019, Vizentin-Bugoni et al., 2021).

### 5.4 Climate change

Climate change is a growing threat to global biodiversity (Bellard et al., 2012). By rapidly altering temperature and precipitation patterns to which species are adapted, climate change is impacting the historic distributional ranges of species, forcing them to migrate altitudinally (latitudinal movements not being an option on most islands) or to adapt to their new climate (Harter et al., 2015). Migration to higher elevations in order to find favorable conditions in which to live may be impossible for some species, depending on island elevation (Harter et al., 2015) and habitat loss. A growing body of studies now provides evidence that climate change poses a serious threat to endemic island biota such as mammals (Leclerc et al., 2020), conifers (Rosenblad et al., 2019), amphibians (Alcala et al., 2012) and bryophytes (Patiño et al., 2016). Another important consequence of climate change is sea-level rise, with its dramatic consequences for terrestrial species (Bellard et al., 2014a, Bellard et al., 2014b), as well as nesting seabirds and marine turtles (Wetzel et al., 2013). Although the loss of terrestrial biodiversity due to the submersion of atolls will not be large in terms of global extinctions (because atolls support few endemics), this has huge socio-economic and cultural consequences; there are 136 atolls in Polynesia, 92 in Micronesia and 66 in Melanesia in the Pacific Ocean alone (Bryan, 1953). Both low and high islands are predicted to suffer from an increase in the frequency and intensity of tropical cyclones as a result of climate change (Chu et al., 2020).

One concern related to climate change is how it will affect the orographic cloud layers which are of great importance for many islands. Orographic cloud layers create, in many high islands worldwide, humid refugia where forested ecosystems can withstand the year-long or seasonal aridity of the macroclimate. Prominent examples include the laurel forest in the Canaries, or the montane cloud forests of the Hawaiian Islands and La Réunion. The altitudinal location, frequency and depth of the cloud layer are also expected to be affected by climate change, though cloud layer projections are still uncertain, with some postulating its altitudinal ascent (Still et al., 1999) and others its descent (Sperling et al., 2004). The ascent in elevation may pose serious risks on islands that have a cloud layer only around their summits, because they may lose it; on the other hand, the descent of the cloud layer’s base means that it will occupy areas in large part already transformed for agriculture or settlements, thus impeding the relocation of the communities for which the cloud layer serves as a climatic refugium.

### 5.5 Synergism between different drivers of biodiversity change

An example from the Hawaiian Islands illustrates how multiple drivers of biodiversity change can act synergistically to produce a ‘perfect storm’ of threats to insular species. Many endemic Hawaiian forest birds began to decline shortly after the arrival of Polynesian explorers about 1000 years ago: land clearance, collection of birds for feathers, and predation by the invasive Pacific rat (*Rattus exulans*) all took a toll. Anthropogenic change associated with European colonization accelerated habitat loss and greatly expanded the suite of mammalian predators. It also introduced novel diseases and disease vectors, including mosquitoes carrying avian malaria, which contributed to the elimination of many native bird species from the lowlands. Together, this has already driven 78 described and undescribed endemic species and subspecies of birds extinct, including 40 of the 58 species of the iconic Hawaiian honeycreepers’ radiation, and all seven species of the endemic family Mohoidae (Hume, 2017; J.P. Hume pers. comm.). Furthermore, recent models have demonstrated how climate change will allow mosquitoes to spread into the last upland refugia of the Hawaiian honeycreepers on Kauaʻi, thereby exposing them to avian malaria, and likely leading to their extinction (Benning et al., 2002, Fortini et al., 2015). To further complicate matters, Hawaiian forests are currently threatened with collapse as a result of a highly virulent, introduced fungal disease that is spreading rapidly and devastating the dominant tree species, *Metrosideros polymorpha*, on which many forest birds depend (Fortini et al., 2019).

### 5.6 Extinction debts

Species extinctions that will happen in the future as a result of past impacts are known as extinction debts. The extinction debt of insular biota owing to anthropogenic habitat loss has been calculated for some Macaronesian archipelagos (Triantis et al., 2010 for Azores; Otto et al., 2017 for the Canaries) and produced concerning perspectives, with a big proportion of the biota of several ecosystems likely carrying huge extinction debts. Conservation efforts targeted at threatened insular species, although indispensable for their short-term survival, will only postpone their final extinction unless lost habitats are restored to sufficient extents. The complete recovery of all lost habitats is neither possible nor desirable, because island societies need to modify landscapes in which to satisfy their economic necessities (agriculture, pastures, infrastructure, settlements, tourist resorts, etc.). However, many disturbed habitats are nowadays abandoned, and the ecological restoration of such sites would provide not only an opportunity to avoid paying the extinction debt to the species affected, but could also contribute to restoring lost ecosystem functions and help mitigate global climate change by fixing atmospheric CO_2_ as biomass (Aronson et al., 2020).

### 5.7 Loss of biotic interactions and ecological functions

With the extinction of a species, a whole suite of biotic interactions (pollination, dispersal, herbivory, or predation) and/or biogeochemical roles or functions (nitrogen fixation, decomposition, nutrient cycling, habitat and resource provisioning, etc.) that the extinct species provided to the ecosystem disappear as well. Some species not directly affected by human impact can thus lose an indispensable interaction partner and vanish, in a process known as a trophic cascade (such as the demise of the Haast eagle (*Harpagornis moorei*) after moa (Dinornithidae) were hunted to extinction in New Zealand). One consequence of the extinction of large native frugivores is the disruption of seed dispersal, altering successional trajectories and shifting contemporary forests away from the pre-human communities rich in fleshy fruited species, as Albert et al., 2020b, Albert et al., 2021 have demonstrated for La Réunion island.

One way to recover the lost roles or functions in the ecosystem is via rewilding, i.e., through introduction of a related taxon, functionally analogous to the missing one. Controlled rewilding experiments are being developed on several islets off Mauritius where extinct giant tortoises (*Cylindraspis* spp.) have been successfully replaced by the Aldabra giant tortoise (*Aldabrachelys*), enhancing seed dispersal and thereby the recovery of native trees that were left without their main disperser after its extinction (Griffiths et al., 2010). Nevertheless, in many cases, the diversity and ecological functions of the extinct native fauna have been replaced by widespread, introduced, generalist species. For example, in the Hawaiian Islands, most native frugivores are rare or extinct and nearly all vertebrate-mediated seed dispersal is carried out by invasive, alien birds (Chimera and Drake, 2010, Vizentin-Bugoni et al., 2019, Vizentin-Bugoni et al., 2021). Similarly, on many oceanic islands, roughly 90% of the endemic, seed-eating vertebrates have been replaced by globally invasive species of birds, rodents, and pigs (Carpenter et al., 2020). What remains to be demonstrated, in most cases, is the extent to which the introduced generalists that occupy the niches of the missing native species actually produce similar outcomes for the native plants with which they interact.

## 6 Insular extinction statistics

**Fig. 4:**
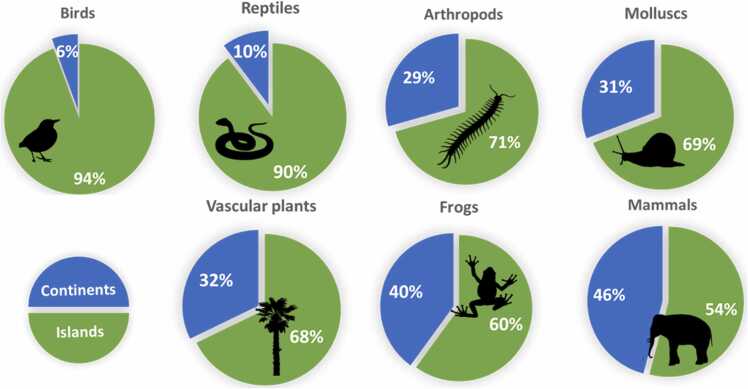
Number of terrestrial species of major taxa that have gone extinct globally, or become extinct in the wild, along with percentages of insular extinctions relative to the total amount of known post-description extinct species.

Source: IUCN, 2017.

**Fig. 5:**
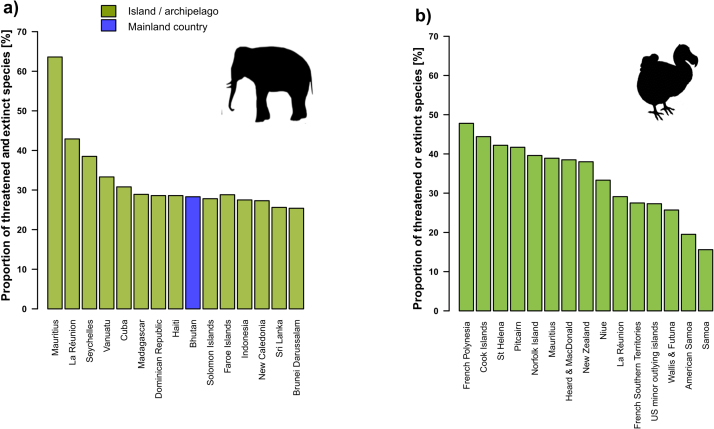
Countries or territories with the highest percentage of threatened and extinct mammal (a) and bird (b) species worldwide. All but Bhutan are islands or archipelagos. Only countries with more than 10 species are included.

Source: Vié et al. (2009).

The disproportionate contribution of islands to global biodiversity noted above is surpassed by islands’ contribution to globally threatened or extinct species (Fig. 1). Roughly 50% of the species recognized as threatened in any of the IUCN threat categories are insular species, and this contribution expands to around 75% of the approximately 800 known extinctions that have occurred since the European expansion around the world (IUCN, 2017). This pattern is consistent among insular taxa, all of them contributing > 50%: birds (94.4%), reptiles (89.7%), arthropods (70.6%), snails (69.2%), vascular plants (67.8%), frogs (60%) and mammals (54.1%) (Fig. 4). To put it in another way, the likelihood of an insular species being driven to extinction by humans is 12 times higher than that of a continental one. Fig. 5 summarizes the 15 countries or territories with the highest percentage of either threatened or extinct species of birds and mammals. Of the 30 geographical entities listed, only one (Bhutan) is continental (Vié et al., 2009).

Furthermore, many island species went extinct due to human activities (such as hunting, predation by introduced herbivores or carnivores, or habitat transformation) long before being recorded and described by European scientists, and are known only from (sub)fossil remains (pre-description extinctions) (e.g., Steadman, 2006). If such extinctions, not considered by IUCN, are added, island extinctions would increase by at least an order of magnitude. For instance, Johnson et al. (2017) quantified the contribution of islands to Late Quaternary (last 40 Ky BP) anthropogenic pre-description species extinctions of endemic vertebrate genera at 57.8%. Slavenko et al. (2016) noted that of the 82 known reptile extinctions that occurred globally over the last 50,000 years, 73 (89%) occurred on islands. Furthermore, of the 268 known mammalian extinctions that occurred during the Holocene, 225 (84%) occurred on islands (Turvey, 2009). Similarly, Duncan et al. (2013) calculated that human colonization of remote Pacific islands caused the global extinction of close to 1000 species of non-passerine land-birds alone; seabird and passerine extinctions would add to this total. Extinctions occurred in multiple waves, beginning with the initial colonization by humans and becoming amplified with subsequent human immigration (Wood et al., 2017, Johnson et al., 2017, Fig. 6).

**Fig. 6:**
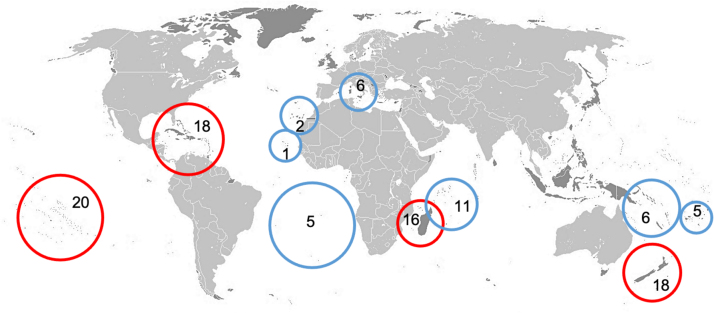
The number of endemic vertebrate genera whose extinction is attributed to human activities across different archipelagos worldwide. In red, those extinctions attributable to pre-European colonizers (pre-description extinctions) and in blue, those attributable to Europeans (post-description extinctions) (based in Johnson et al., 2017).

Map adapted from https://commons.wikimedia.org/ with dark grey areas indicating islands.

Following Turvey (2009) pre-European extinctions of charismatic species include 17 Madagascan giant lemurs (Lemuridae), four elephant birds (Aepyornithidae), and three pigmy hippopotamuses (*Hyppopotamus* spp.), ten New Zealand moa (Dinornithiformes), numerous Hawaiian honeycreepers (Drepanidinae), crows (Corvidae), and flightless nēnē nui and moa nalo (Anseriformes), the Balearic mouse-goat (*Myotragus balearicus*), several giant Caribbean sloths (Megalocnidae), the Cuban giant solenodon (*Solenodon arredondoi*), as well as the Canarian giant lizard (*Gallotia goliath*) (Castillo et al., 1994), and the Easter Island palm (*Paschalococos disperta*) (Marchant et al., 2009).

Post-European-contact extinctions include several Mascarene *Cylindraspis* tortoises, the Mauritius dodo (*Raphus cucullatus*), the Rodrigues solitaire (*Pezophaps solitaria*), the Réunion ibis (*Threskiornis solitarius*) (Cheke and Hume, 2008), the Commander Islands (Steller’s) sea cow (*Hydrodamalis gigas*) (Anderson, 1995), roughly 30 species of *Partula* snails endemic to Society Islands (Coote and Loéve, 2003), several St. Helena arboreal sunflowers (Asteraceae) (Cronk, 1989) and more recently the Canarian black oystercatcher (*Haemotopus meadewaldoi*) (Valledor de Lozoya, 2013), the Cabo Verde giant skink (*Chioninia coctei*) (Rocha et al., 2015) and the Tasmanian thylacine (*Thylacinus cynocephalus*) (Paddle, 2002), just to mention some of the more spectacular cases. Bird extinctions in the Hawaiian Islands have continued into the 21st century (Hume, 2017). Simultaneously, many other inconspicuous species, less well known but undoubtedly playing important roles in their natural ecosystems, have also been driven extinct by human activities on islands, including many vascular plants, arthropods, mollusks and other invertebrates.

All these examples illustrate why, within the 35 biodiversity hotspots identified by *Conservation International* (2005) (which cover just 2.3% of the Earth’s surface and are home to over the 50% of the world’s plant species and 42% of terrestrial vertebrates), nine hotspots are composed exclusively of islands: (1) the Caribbean islands, (2) Madagascar and adjacent islands (the Comoros, Mascarenes, and Seychelles), (3) East Melanesia, (4) Japan, (5) New Caledonia, (6) New Zealand, (7) the Philippines, (8) Polynesia-Micronesia, and (9) Wallacea. Three additional hotspots have a substantial proportion of their diversity and endemism within islands: (10) the Mediterranean basin (including Macaronesia), (11) the Western Ghats and Sri Lanka and (12) Sundaland (including the large land-bridge islands of Sumatra, Borneo and Java). Conversely, New Guinea—the world's largest tropical island, harboring the richest island flora with almost 14,000 plant species, of which 68% are endemic (Cámara-Leret et al., 2020)—is traditionally considered as a wilderness area, a status that should be reconsidered due to increasing encroachment, mining, oil palm expansion, and construction of major new infrastructures like the Trans-Papua highway (Shearman et al., 2009).

## 7 An uncertain future for islands in the Anthropocene

The data summarized in this paper are overwhelmingly clear: as they have for centuries, islands continue to bear the brunt of the biodiversity crisis. Unfortunately, the slope of the extinction curve that began with the arrival of the first human voyagers and continued with the later waves of colonization has become even steeper in recent decades (Whittaker et al., 2017), meaning that we are still too far from bending this curve. Immediate action embraced by a consensus of policy makers, scientists, environmentalists, and society is urgently needed if island extinctions are to be halted, and healthy island environments conserved and restored (Box 1).

Box 1 **Recommended actions to stop further loss of biodiversity and to assist in the recovery of threatened island biota.**

Islands have suffered disproportionately high levels of species extinctions and habitat degradation, and today they are home to a high number of the world’s threatened species. It is imperative that actions be taken to stop the further loss of biodiversity, and to assist in the recovery of threatened island biota. We recommend action in several areas:

**Increase knowledge:**

Fund scientific surveys for completing the biotic inventory of understudied islands (Guinea Gulf, Red Sea, Andaman, Nicobar, Micronesia, Desventuradas, etc.) and taxa (fungi, amoeba, Acari, Collembola, taxonomically difficult insects (Diptera, Hymenoptera), mycorrhizae, nematodes, flatworms, etc.) to create baseline knowledge for future monitoring initiatives.

Implement long-term monitoring projects with adequate standardized sampling protocols (e.g. Borges et al., 2018, Borges et al., 2020).

**Conserve threatened species:**

Evaluate all island endemic species for IUCN Red List status, to aid in establishing conservation priorities (see Hochkirch et al., 2021).

Develop *ex situ* conservation programs (e.g., captive breeding/propagation or germplasm banks) for appropriate threatened species.

**Manage invasive alien species (IAS):**

Develop lists of problematic IAS and biosecurity measures to restrict their spread.

Develop and implement techniques to control or eradicate problematic IAS.

**Protect remaining habitat:**

Establish a minimum threshold of 30% of the area occupied by each habitat type in each island group for its protection, in accordance with the Post 2020 Global Biodiversity Framework of the CBD https://www.unep.org/resources/publication/1st-draft-post-2020-global-biodiversity-framework.

Establish corridors to connect habitat types and allow dispersal and altitudinal migration in response to disturbances and climate change.

**Restore degraded habitat**:

Promote habitat restoration for halting the extinction debt and for capturing CO_2_ in biomass.

Establish predator-free islands or exclosures to allow reassembly of native communities.

Reintroduce extirpated species to areas from which threats have been eliminated.

Where appropriate, apply synthetic biology and/or rewilding techniques to address loss of island biodiversity.

Evaluate established non-native species as potential surrogates for extinct species (in novel ecosystems).

**Build local conservation capacity:**

Assist island communities to conserve their natural heritage by providing resources and technical support.

Develop a regular forum for sharing information among the conservation scientists and managers who are isolated among the world’s far flung islands.

**Support island communities as stewards of global biodiversity:**

Develop sustainable living conditions for island communities to allow nature and humans to thrive together.

Find public and private international donors engaged with the conservation of insular biota.

Conservation of threatened island biota is still possible; there are some positive and encouraging conservation messages coming from different islands around the world. For example, 65% of the bird species whose extinction has been prevented by conservation actions over the past three decades were confined to islands (Bolam et al., 2020). Islands have also been subjected to over 700 successful eradication programs of alien vertebrates (among them mice, rats, rabbits, goats, pigs, and cats) (Keitt et al., 2011). Additionally, ambitious long-term conservation projects, such as Predator Free 2050, (2018) or Auwahi forest restoration project (1997) have been established. Unfortunately, the rate of species introductions on islands surpasses by several orders of magnitude the rate of successful eradications, most of which are restricted to very tiny islands. And in some cases, allocation of conservation resources for island species lags behind that for continental species; for instance, endangered birds on the US mainland receive roughly fifteen times as much funding per species as endangered Hawaiian birds (Leonard, 2008). Also, many small island nations lack the resources or infrastructure to mount conservation campaigns at the required scale.

One in four of the world’s nations are insular, implying that small island states could form an important political lobby to advocate for island conservation in appropriate forums (such as Convention on Biological Diversity, United Nations Environmental Program, UNESCO, etc.). Island conservation initiatives should involve local communities, NGOs, managers and authorities, especially in remote or isolated islands (e.g., in the South Pacific) to ensure long-term monitoring and sustainability. With that aim, it is crucial to train and support local island conservation biologists because, independent of the arrival of funding from international donors, they represent the best guarantee of a future for the insular biota.

Since 2014 a series of international Symposia on Island Biology (Honolulu in Hawaiʻi, 2014; Angra do Heroismo in Azores, 2016; St. Denis in La Réunion, 2019) have taken place with the participation of ca. 400 scientists and conservation professionals in each—experts that work on different topics and in different island groups worldwide. In the most recent symposium, these scientists pledged to constitute the Society of Island Biology, SIB (https://islandbiology.com/) a scientific society that aims to bring together researchers and conservation managers who, by the very nature of the islands they study, face geographical barriers to communication and collaboration. In addition to its academic, scientific goals, SIB is committed to providing a recognizable voice to the collective of world island biologists so that they can be heard in those forums where decisions concerning the biodiversity of islands and archipelagos are taken, and to advocate for the conservation of the biodiversity of islands in all those ambits where its survival may be in peril. With this warning, the authors of this article, and SIB members representing the Island Biology researchers worldwide, would like to contribute to these noble goals.

## Declaration of Competing Interest

The authors declare that they have no known competing financial interests or personal relationships that could have appeared to influence the work reported in this paper.
