# Supplementary material for: Scientists’ warning – The outstanding biodiversity of islands is in peril
Source: Glob Ecol Conserv. 2021 Nov;31:e01847. doi: 10.1016/j.gecco.2021.e01847 (PMC8556160; doi:10.1016/j.gecco.2021.e01847)
Supplement: Supplementary file 1 — Supplementary material [file mmc1.pdf]

Supporting Information

List of references used in Table 1:

Alonso, M.R., Ibañez, M., 2015. The species of family Enidae B. B. Woodward, 1903 (1880) (Mollusca, Gastropoda, Stylommatophora) from the Canary Islands: the genus *Napaeus* Albers, 1850. Vieraea 43, 153–188.

Arechavaleta, M., Zurita, N., Marrero, M.C., Martín, J.L., (Eds.), 2005. Lista preliminar de especies silvestres de Cabo Verde. Hongos, plantas y animales terrestres. 2005. Gobierno de Canarias.

Arechavaleta, M., Rodríguez, S., Zurita, N., García, A., (Eds.), 2010. Lista de especies silvestres de Canarias. Hongos, plantas y animales terrestres. 2009. Gobierno de Canarias.

Arensburger, P., Buckley, T.R., Simon, C., Moulds, M., Holsinger, K.E., 2004. Biogeography and phylogeny of the New Zealand cicada genera (Hemiptera: Cicadidae) based on nuclear and mitochondrial DNA data. J Biogeogr 31, 557–569. https://doi.org/10.1046/j.1365-2699.2003.01012.x

Arnedo, M.A., Oromí, P., Ribera, C., 2000. Systematics of the Genus *Dysdera* (Araneae, Dysderidae) in the Eastern Canary Islands. J Arachnol 28, 361–292.

Baldwin, B.G., Sanderson, M.J., 1998. Age and rate of diversification of the Hawaiian silversword alliance (Compositae). Proc Natl Acad Sci USA 95, 9402–9406. https://doi.org/10.1073/pnas.95.16.9402

Barrabé, L., Maggia, L., Pillon, Y., Rigault, F., Mouly, A., Davis, A.P., Buerki, S., 2014. New Caledonian lineages of *Psychotria* (Rubiaceae) reveal different evolutionary histories and the largest documented plant radiation for the archipelago. Mol Phylogenet Evol 71, 15–35. https://doi.org/10.1016/j.ympev.2013.10.020

Bayly, M., Kellow, A., 2006. An illustrated guide to New Zealand Hebes. Te Papa Press, Wellington.

Borges, P.A.V., Abreu, C., Aguiar, A.M.F., Carvalho, P., Jardim, R., Melo, I., Oliveira, P., Sérgio C., Serrano, A.R.M., Vieira, P., 2008. Listagem dos fungos, flora e fauna ter- restres dos arquipélagos da Madeira e Selvagens. Direcção Regional do Ambiente da Madeira and Universidade dos Açores, Funchal y Angra do Heroísmo, Portugal.

Borges, P.A.V., Amorim, I. R., Terzopoulou, S., Rigal, F., Emerson, B.C., Serrano, A.R.M., 2017. Cryptic diversity in the Azorean beetle genus *Tarphius* Erichson, 1845 (Coleoptera: Zopheridae): An integrative taxonomic approach with description of four new species. Zootaxa 4236, 401–449. https://www.biotaxa.org/Zootaxa/article/view/zootaxa.4236.3.1

Borges, P.A.V., Costa, A., Cunha, R., Gabriel, R., Gonçalves, V., Martins, A.F., Melo, I., Parente, M., Raposeiro, P., Rodrigues, P., Santos, R.S., Silva, L., Vieria, P., Vieria, V., (Eds.) 2010. A list of the terrestrial and marine biota from the Azores. Principia, Cascais.

Bueanvista, D.P., 2017. Contributions to the orchid flora of Mindanao. Long-term ecological research sites, Philippines. Biologica Nyssana 8, 31–38. http://journal.pmf.ni.ac.rs/.../166.

Callmander, M.W., Laivao, M.O., 2003. New findings on *Pandanu*s sect. *Imerinenses* and sect. *Rykiella* (Pandanaceae) from Madagascar. Adansonia 25, 53–63.

https://sciencepress.mnhn.fr/en/periodiques/adansonia/25/1/nouvelles-donnees-sur-le-genre-pandanus-sect-imerinenses-et-sect-rykiella-pandanaceae-de-madagascar

Carvalho, J.A, Pontes, T., Batista-Marques, M.I., Jardim, R., 2010. A new species of *Echium* (Boraginaceae) from the island of Porto Santo (Madeira Archipelago). Anales del Jardín Botánico de Madrid 67, 87–96. https://doi.org/10.3989/ajbm.2239.

Chambers, S.M., 1991. Biogeography of Galápagos land snails, in: James, M.J., (Ed.), Galápagos Marine Invertebrates, New York: Plenum. pp, 307–25.

Chiba, S., 1999. Accelerate evolution of land snails *Mandarina* in the oceanic Bonin islands: evidence from mitochondrial DNA sequences. Evolution 53, 460–471. https://doi.org/10.1111/j.1558-5646.1999.tb03781.x

Cho, M.S., Yang, J.Y., Yang, T.J., Kim, S.C., 2019. Evolutionary comparison of the chloroplast genome in the woody *Sonchus* Alliance (Asteraceae) on the Canary Islands. Genes 10, 217. https://doi.org/10.3390/genes10030217

Cotoras, D.D., Bi, K, Brewer, M.S., Lindberg, D.R., Prost, S., Gillespie, R.G., 2018. Co-occurrence of ecologically similar species of Hawaiian spiders reveals critical early phase of adaptive radiation. BMC Evol Biol 18, 100. https://bmcecolevol.biomedcentral.com/articles/10.1186/s12862-018-1209-y#:~:

Crespo, L.C., Silva, I., Enguídanos, A., Cardoso, P., Arnedo, M.A., 2020. Integrative taxonomic revision of the woodlouse-hunter spider genus *Dysdera* (Araneae: Dysderidae) in the Madeira archipelago with notes on its conservation status. Zool J Linn Soc 192, 356–415. https://doi.org/10.1093/zoolinnean/zlaa089.

Dransfield, J., Beentje, H.J., 1995. Palms of Madagascar. Royal Botanical Gardens Kew and the International Palm Society.

Dransfield, J., Uhl, N.W., Asmussen, C.B., Baker, W.J., Harley, M.M., Lewis, C., 2008. Genera Palmarum. The evolution and classification of Palms. Kew Publishing. https://kew.iro.bl.uk/concern/books/503db94e-b77d-4f40-8707-1d1b602b2b86?locale=en.

Emerson, B.C., Oromí, P., 2005. Diversification of the forest beetle genus *Tarphius* on the Canary Islands, and the evolutionary origins of island endemics. Evolution 59, 586–598. https://doi.org/10.1111/j.0014-3820.2005.tb01018.x.

Fernández-Mazuecos, M., Vargas. P., McCauley, R.A. Monjas, D. Otero, A., Chaves, J.A., Guevara Andino, J.E., Rivas-Torres, G., 2020. The radiation of Darwin's Giant Daisies in the Galápagos Islands. Current Biology 30, 1–10. https://doi.org/10.1016/j.cub.2020.09.019.

García-Maroto, F., Mañas-Fernández, A., Garrido-Cárdenas, J.A., Alonso, D.L., Guil-Guerrero, J.L., Guzmán, B., Vargas, P., 2009. Δ6-Desaturase sequence evidence for explosive Pliocene radiations within the adaptive radiation of Macaronesian *Echium* (Boraginaceae). Mol Phylogenet Evol 52, 563–574. https://doi.org/10.1016/j.ympev.2009.04.009.

Givnish, T.J., Millam, K.C., Mast, A.R., Paterson, T.B., Theim, T.J., Hipp, A.L., Henss, J.M., Smith, J.F., Wood, K.R., Sytsma, K.J., 2009. Origin, adaptive radiation and diversification of the Hawaiian lobeliads (Asterales: Campanulaceae). P Roy Soc B 276, 407–416. https://doi.org/10.1098/rspb.2008.1204.

Grant, P., Grant, R., 2008. How and why species multiply. The radiation of Darwin's finches. Princeton University Press, Princeton. https://press.princeton.edu/books/paperback/9780691149998/how-and-why-species-multiply.

Groom, S.V.C., Stevens, M.I., Schwarz, M.P., 2013. Diversification of Fijian halictine bees: insights into a recent island radiation. Mol Phylogenet Evol 68, 582–594. https://doi.org/10.1016/j.ympev.2013.04.015.

Heads, M., 2001. Birds of Paradise, biogeography and ecology in New Guinea: A review. J Biogeogr 28, 893–925. https://doi.org/10.1046/j.1365-2699.2001.00600.x.

Heads, M., 2006. Seed plants of Fiji: an ecological analysis. Biol J Linn Soc 89, 407–431. https://doi.org/10.1111/j.1095-8312.2006.00682.x.

Heaney, L.R., Balete, D.S., Rickart, E.A., 2016. The mammals of Luzón island. Biogeography and Natural History of a Philippine fauna. Johns Hopkins University Press, Baltimore. https://jhupbooks.press.jhu.edu/title/mammals-luzon-island.

Herrera, J.P., 2017. Testing the adaptive radiation hypothesis for the lemurs of Madagascar. Roy Soc Open Sci 4, 161014. https://doi.org/10.1098/rsos.161014.

Jønsson, K.J., Fabre, P.H., Fritz, S.A., Etienne, R.S., Ricklefs, R.E., Jørgensen, T.B., Fjeldså, J., Rahbek, C., Ericson, G.P., Woog, F., Pasquet, E., Irested, M., 2012. Ecological and evolutionary determinants for the adaptive radiation of the Madagascan vangas. Proc Natl Acad Sci USA 109, 6620–6625. https://doi.org/10.1073/pnas.1115835109.

Kitson, J.N., Warren, B.H., Florens, F.B.V., Baider, C., Strasberg, D., Emerson, B.C., 2013. Molecular characterization of trophic ecology within an island radiation of insect herbivores (Curculionidae: Entiminae: *Cratopus*). Mol Ecol 22, 5441–5455. https://doi.org/10.1111/mec.12477.

Kleinkopf, J.A., Roberts, W.R., Wagner, W.L., Roalson, E.H., 2019. Diversification of Hawaiian *Cyrtandra* (Gesneriaceae) under the influence of incomplete lineage sorting and hybridization. J Syst Evol 57, 561–578. https://doi.org/10.1111/jse.12519

Knope, M.L., Funk, V.A., Johnson, M.A., Wagner, W.L., Datlof, E.M., Johnson, G., Crawford, D.J., Bonifacino, J.M., Morden, C.M., Lorence, D.H., Wood, K.E., Meyer, J.I., Carlquist, S., 2020. Dispersal and adaptive radiation of *Bidens* (Compositae) across the remote archipelagoes of Polynesia. J Syst Evol 58, 805–822. https://doi.org/10.1111/jse.12704.

Le Péchon, T. Humeau, L., Gigord, L. Pause, J.B., Caron, D., Baider, C., Gigord, P., Grosser, D., Hansen, D., Sevathian, J.C., 2013. Les Mahots des Mascareignes. Base de connaissances sur les Dombeyoideae des Mascareignes. Univ. La Réunion, St Denis. https://cel.archives-ouvertes.fr/LIM/hal-01186487.

Lee, T., Li, J., Churchill, C.K.C., Foighil, D.O., 2014. Evolutionary history of a vanishing radiation: isolation-dependent persistence and diversification in Pacific Island partulid tree snails. BMC Evol Biol 14, 202. https://bmcecolevol.biomedcentral.com/articles/10.1186/s12862-014-0202-3

Liebherr, J.K., 2013. The *Mecyclothorax* beetles (Coleoptera, Carabidae, Moriomorphini) of Tahiti, Society Islands. ZooKeys 32, 1–170. https://doi.org/10.3897/zookeys.224.3675

Losos, J.B., 2009. Lizards in an evolutionary tree: ecology and adaptive radiation of anoles. University California Press, Berkeley, CA. https://www.ucpress.edu/book/9780520269842/lizards-in-an-evolutionary-tree.

Machado, A., Rodríguez-Expósito, E., López, M., Hernández, M., 2017. Phylogenetic analysis of the genus *Laparocerus*, with comments on colonisation and diversification in Macaronesia (Coleoptera, Curculionidae, Entiminae). ZooKeys 651, 1–77. https://doi.org/10.3897/zookeys.651.10097. eCollection 2017.

Macías-Hernández, N., de la Cruz López, S., Roca-Cusachs, M., Oromí, P., Arnedo, M.A., 2016. A geographical distribution database of the genus *Dysdera* in the Canary Islands (Araneae, Dysderidae). ZooKeys 625, 11–23. https://doi.org/10.3897/zookeys/625.9847.

Magnacca, K.N., 2007. Conservation status of the endemic bees of Hawai‘i, *Hylaeus*, (*Nesoprosopis*) (Hymenoptera: Colletidae). Pac Sci 61, 173–190. http://hdl.handle.net/10125/22606.

Munzinger, J., Mora,T.P., Jaffré, T., Gâteblé, G., Pillon, Y., Tronchet, F., Veillon, J.-M., Chalopin M., 2016. FLORICAL: Checklist of the vascular indigenous flora of New Caledonia. http://publish.plantnet-project.org/project/florical

Nielsen, S.V., Bauer, A.M., Jackman, T.R., Hitchmough, R.A., Daugherty, C.H., 2011. New Zealand geckos (Diplodactylidae): Cryptic diversity in a post-Gondwanan lineage with trans-Tasman affinities. Mol Phylogenet Evol 59, 1–22. https://doi.org/10.1016/j.ympev.2010.12.007

O'Grady, P., DeSalle, R., 2018. Hawaiian *Drosophila* as an evolutionary model clade: days of future past. BioEssays 40, 1700246. https://doi.org/ 10.1002/bies.201700246

Paulay, G., 1985. Adaptive radiation on an isolated oceanic island: the Cryptorhynchinae (Curculionidae) of Rapa revisited. Biol J Linn Soc 26, 95–187. https://doi.org/10.1111/j.1095-8312.1985.tb01554.x.

Pratt, H.D., 2005. The Hawaiian honeycreepers. Oxford University Press, NY. https://global.oup.com/academic/product/the-hawaiian-honeycreepers-9780198546535?cc=es&lang=en&.

Rivera, J.O., Kraus, F., Allison, A., Butler, M.A., 2017. Molecular phylogenetics and dating of the problematic New Guinea microhylid frogs (Amphibia: Anura) reveals elevated speciation rates and need for taxonomic reclassification. Mol Phylogenet Evol 112, 1–11. https://doi.org/10.1016/j.ympev.2017.04.008.

Sang, T., Crawford, D.J., Kim, S.C., Stuessy, T.F., 1998. Radiation of the endemic genus *Dendroseris* (Asteraceae) on the Juan Fernández Islands: evidence from sequences of its regions of nuclear ribosomal DNA. Am J Bot 81, 1494–1501. https://doi.org/10.2307/2445322.

Sarnat, E.M., Hita Garcia, F., Dudley, K., Liu, C., Fischer, G., Economo, E.P., 2019. Ready species one: exploring the use of augmented reality to enhance systematic biology with a revision of Fijian *Strumigenys* (Hymenoptera: Formicidae). Insect Systematics and Diversity 3, 1–43. https://doi.org/10.1093/isd/ixz005.

Shaw, K.L., 1995. Biogeographic patterns of two independent Hawaiian cricket radiations. (*Laupala* and *Prognathogryllus*), in: Wagner, W., Funk, V.A., (Eds.) Hawaiian Biogeography: Evolution on a hot spot archipelago. Smithsonian, Washington. pp. 39–56.

Skema, C., 2012.Toward a new circumscription of *Dombeya* (Malvales: Dombeyaceae): A molecular phylogenetic and morphological study of *Dombeya* of Madagascar and a new segregate genus, *Andringitra*. Taxon 61, 612–628. https://doi.org/10.1002/tax.613010

Skipwith, P.L., Bi, K., Oliver, P.M., 2019. Relics and radiations: Phylogenomics of an Australasian lizard clade with east Gondwanan origins (Gekkota: Diplodactyloidea). Mol Phylogenet Evol 140, 106589. http://doi.org/10.1016/j.ympev.2019.106589.

Smith, S.A., Sadlier, R.A., Bauer, A.M., Austin, C.C., Jackman, T., 2007. Molecular phylogeny of the scincid lizards of New Caledonia and adjacent areas: Evidence for a single origin of the endemic skinks of Tasmantis. Mol Phylogenet Evol 43, 1151–1166. https://doi.org/10.1016/j.ympev.2007.02.007.

Strijk, J., Noyes, R.D., Strasberg, D., Cruaud, C., Gavory, F., Chase, M.W., Abbott, R.J., Thébaud, C., 2012. In and out of Madagascar: Dispersal to Peripheral Islands, Insular Speciation and Diversification of Indian Ocean Daisy Trees (*Psiadia*, Asteraceae). PLoS One 7, e42932. https://doi.org/10.1371/journal.pone.0042932.

Taylor, C.M., 2020. Overview of *Psychotria* in Madagascar (Rubiaceae, Psychotrieae), and of Bremekamp's foundational study of this group. Candollea 75, 51–10. https://doi.org/10.15553/c2020v751a5.

Vollering, J., Schuiteman, A., de Vogel, E., van Vugt, R., Raes, N., 2016. Phytogeography of New Guinean orchids: patterns of species richness and turnover. J Biogeogr 43, 204–214. https://doi.org/10.1111/jbi.12612.

Von Rintelen, T., Glaubrecht, M., 2005. Anatomy of an adaptive radiation: a unique reproductive strategy in the endemic freshwater gastropod *Tylomelania* (Cerithioidea: Pachychilidae) on Sulawesi, Indonesia and its biogeographical implications. Biol J Linn Soc 85, 513–542. https://doi.org/10.1111/j.1095-8312.2005.00515.x.
